# Ergonomic Trends in Endoscopic Skull‐Base Surgeons: A Survey to the North American Skull Base Society

**DOI:** 10.1002/oto2.70220

**Published:** 2026-03-09

**Authors:** Shreya Mandloi, Alexander Duffy, Praneet Kaki, Elina M. Toskala, Marc Rosen, Christopher Farrell, James Evans, Gurston G. Nyquist, Mindy Rabinowitz

**Affiliations:** ^1^ Department of Otolaryngology Thomas Jefferson University Hospital Philadelphia Pennsylvania USA; ^2^ Department of Neurosurgery Thomas Jefferson University Hospital Philadelphia Pennsylvania USA

**Keywords:** endoscopic skull base surgery, musculoskeletal injury, questionnaire, surgical ergonomics

## Abstract

**Objective:**

Lack of ergonomic practices in the operating room results in significant musculoskeletal strain. This study obtains a current assessment of self‐reported musculoskeletal strain associated with endoscopic endonasal and open skull base surgery as reported by members of the North American Skull Base Society (NASBS).

**Study Design:**

Online Survey.

**Setting:**

Online.

**Methods:**

A survey assessing current symptoms in endoscopic skull base surgery was distributed to members of the NASBS.

**Results:**

Sixty‐six responses were completed and analyzed. 95% of respondents indicated they had musculoskeletal strain at the neck and C‐spine. There was no significant difference in average operating time per week and musculoskeletal strain. Prolonged standing during endoscopic cases increased neck/C‐spine musculoskeletal strain (OR: 3.96; CI: 1.26, 14.2; *P* = .024). Dominant hand, operating with 1 versus multiple gloves, switching holding the endoscope with other physicians, foot pedal position, monitor, position, and manipulation of endoscopic instruments did not impact musculoskeletal strain (*P* > .05). Endoscopic endonasal skull base surgeons (EESBs) that operate on the same side of the table had significantly less L‐spine pain than EESBs that operate on the opposite side of the table (OR: 0.25; CI: 0.07, 0.82; *P* = .026).

**Conclusion:**

The results found that most respondents experience musculoskeletal strain and that factors including surgeon positioning and prolonged standing increase pain. Altering strategies in the operating room, including surgeon and instrument positioning, may help to decrease ergonomic strain amongst skull base surgeons.

Surgical ergonomics has been extensively studied within a variety of surgical fields including colorectal, gynecologic, robotic surgery and many others.^1^, ^2^, ^3^, ^4^ Literature has demonstrated that otolaryngology is one of the surgical fields with the highest risk of ergonomic strain due to the use of head‐mounted lighting and loupe magnification confined to a small anatomical surgical field.^5^, ^6^ Moreover, musculoskeletal strain is significantly higher in endoscopic surgery compared to open surgery due to instrument design, the use of monitors and foot pedals, and the positioning of the endoscope.^7^, ^8^, ^9^ As the approach to the anterior skull base has largely shifted from microscopic to endoscopic in the past decade, both neurosurgeons and rhinologists are at risk for increased musculoskeletal strain.^10^ Prior studies have investigated surgical practices amongst skull base surgeons in relation to endoscopic transsphenoidal surgery and have demonstrated that surgeon and patient positioning can influence musculoskeletal strain.^10^ To date, there is a lack of literature investigating surgical practices amongst endoscopic skull base surgeons. The objective of this study was to obtain a current assessment of self‐reported musculoskeletal strain associated with endoscopic endonasal and open skull base surgery as reported by members of the North American Skull Base Society (NASBS).

## Methods

A 43‐question online survey using the RedCap platform was generated by the authors based on prior experience in the operating rooms. The survey received approval by the NASBS and was distributed to members of the NASBS over 3 months in 2024 under Thomas Jefferson Institutional Review Board 11D.10. The survey link was emailed to the entire database of members that are subscribed to the NASBS once a month over 3 months. Only complete responses were analyzed and 6% of the NASBS responded to the survey. The survey was divided into 3 sections: demographics (11 questions), operative information (13 questions), and symptomatology (19 questions). Responses were received over 3 months in 2024 and were anonymous. Survey questions are attached in Supplemental Document S1, available online.

### Statistical Analysis

Continuous variables were reported as mean (standard deviation) and categorical variables were reported as frequency (%). Descriptive statistics were performed using Kruskal‐Wallis rank sum, Pearson chi‐square, and Fisher exact tests, as appropriate, to compare survey outcomes between academic and private physicians, as well as neurosurgeons and rhinologists, respectively. Multiple comparisons were accounted for using the Benjamini‐Hochberg false discovery rate (FDR) correction, and thresholds for significance were adjusted accordingly. Multivariable logistic regression models were used to evaluate factors including glove size, operating room set up, endoscope tool manipulation, monitor and foot pedal position, switching holding the endoscope, fixed posture, and patient habitus that may contribute to musculoskeletal pain. Musculoskeletal strain was defined as rating overall musculoskeletal strain ≥4/10 (Question 3 Symptomatology) or any body part that had ≥4/10 strain (Question 1 Symptomatology). Statistical analysis was performed using R Studio with *P* < .05 for statistical significance.

## Results

### Respondent Demographics

Seventy‐seven total responses were received. Of which, 66 were complete and were analyzed. Most respondents were academic physicians (n = 48, 73%) and there was no significant difference between the number of neurosurgeons (n = 31, 47%) and rhinologists (n = 35, 53%) that answered the survey (*P* = .40). Upon further stratification of the cohort by academic versus private practice endoscopic endonasal skull base surgeons (EESBs), there were no significant differences in years of practice outside of training, average operative hours per week, and percent of operative time using an endoscope (*P* > .05). Academic EESBs routinely operate with residents and fellows significantly more than private EESBs (*P* < .001). Most respondents in both private and academic medicine use both hands to manipulate operative instruments while another person holds the scope (89%). All respondent demographics are detailed in Table 1; and demographic similarities and differences between academic and private EESBs are shown in Table 2. Additionally, demographic differences between neurosurgeons and rhinologists are characterized in Table 3.

**Table 1:** Overall Respondent Demographics

Variable	Average (SD), N (%)
Age	45.9 (10.2)
Gender	
Female	9 (14%)
Male	56 (85%)
Declined to answer	1 (1.5%)
Years in practice	11.8 (10.2)
Operative hours per week	22.9 (7.9)
% of Time utilizing an endoscope	37.1 (21.1)
Specialty	
Neurosurgeon	31 (47%)
Rhinologist	35 (53%)
Practice setting	
Academic	48 (73%)
Private	18 (27%)

**Table 2:** Demographic Characteristics Between Academic and Private Physicians

Variable	Academic cohort average (SD), N (%)	Private cohort average (SD), N (%)	*P*‐value
Age	46.3 (10.2)	44.9 (9.1)	.700
Gender			.600
Female	8 (17%)	1 (6%)	
Male	39 (81%)	16 (94%)	
Declined to answer	1 (2%)	0 (0%)	
Years in practice	12.1 (10.5)	10.9 (9.4)	.800
Operative hours per week	23.7 (8.1)	20.8 (7.0)	.200
% of Time utilizing an endoscope	34.2 (19.4)	45.1 (24.2)	.120
Specialty			.400
Neurosurgeon	21 (43%)	10 (56%)	
Rhinologist	27 (57%)	8 (44%)	
Operate with resident/fellow	44 (92%)	6 (33%)	<.001

**Table 3:** Demographic Characteristics Between Neurosurgeons and Rhinologists

Variable	Neurosurgery cohort average (SD), N (%)	Rhinology cohort average (SD), N (%)	*P*‐value
Age	47.9 (9.0)	44.2 (11.4)	.1
Gender			.027
Female	0 (0%)	9 (26%)	
Male	30 (96%)	26 (74%)	
Declined to answer	1 (2%)	0 (0%)	
Years in practice	12.8 (9.3)	10.7 (11.2)	.3
Operative hours per week	24.7 (8.0)	21.9 (7.7)	.15
% of time utilizing an endoscope	36.2 (20.2)	37.7 (21.2)	.8
Practice type			.4
Academic	21 (67%)	27 (77%)	
Private	10 (3.2%)	8 (33%)	
Operate with resident/fellow	22 (76%)	25 (76%)	.9

### Musculoskeletal Pain Experienced by EESBs—Entire Cohort Analysis

Sixty‐three out of 66 (95%) respondents indicated that they had musculoskeletal pain >4/10 while operating, with the highest strain at the neck/C‐spine (50%) followed by the lower back/L‐spine (42%). Demographic variables including age, BMI, and exercise activity were not correlated with musculoskeletal strain. Average operating time per week was not significantly correlated with musculoskeletal strain. Similarly, factors including dominant hand, operating with one versus multiple gloves, switching holding the endoscope with other physicians, foot pedal position, monitor, position, manipulation of endoscopic instruments, and patient habitus did not impact musculoskeletal strain (*P* > .05) for any body part. Prolonged standing during endoscopic cases was associated with an increased risk of experiencing neck/C‐spine musculoskeletal strain (OR: 3.96; CI: 1.26, 14.2; *P* = .024). During endoscopic resection, EESBs that place one instrument in the same nostril below the endoscope and other instrument in the opposite nostril from the endoscope were less likely to experience musculoskeletal strain compared to physicians that place all of their instruments in the opposite nostril of the endoscope (OR: 0.27; CI: 0.07, 0.90, *P* = .038) (Operative Information Question Number 8). Additionally, neurosurgeons and rhinologists that operate on the same side of the table were less likely to experience L‐spine pain than neurosurgeons and rhinologists that operate on opposite sides of the table (OR: 0.25; CI: 0.07, 0.82; *P* = .026). EESBs that operate with the rhinologist at the head and the neurosurgeon on the patient's side had similar risk of experiencing musculoskeletal strain compared to EESBs that operate with rhinologists and neurosurgeons on opposite sides of the table (OR: 1.11; CI: 0.24, 5.58; *P* = .900) (Operative Information Question Number 7). Factors that impact musculoskeletal strain for the cohort are shown in Table 4.

**Table 4:** Analysis of Factors That Influence Musculoskeletal Strain for EESBS

	Neck/C‐Spine		Low back/L‐Spine		MSK strain > 4/10	
Variable	Odds ratio (CI)	*P*‐value	Odds ratio (CI)	*P*‐value	Odds ratio (CI)	*P*‐value
Age	0.16 (0.01, 1.78)	.13	0.17 (0.01, 1.88)	.22	0.14 (0.01, 1.59)	.11
Height	2.2 (0.76, 4.0)	.668	2.1 (0.50, 5.23)	.988	2.10 (0.76, Inf)	.429
Weight	1.2 (0.98, 1.05)	.9	1.3 (0.7, 1.7)	.8	1.01 (0.98, 1.05)	.5
**Prolonged standing**	**3.96 (1.26, 14.2)**	**.024**	1.2 (0.40, 3.73)	.8	1.03 (0.32, 3.39)	.99
Manipulation of endoscopic instruments	3.31 (0.72, 19.20)	.14	0.25 (0.04, 1.09)	.09	1.70 (0.39, 7.39)	.5
Foot pedal position	0.96 (0.14, 6.57)	.9	1.64 (0.29, 9.27)	.6	0.76 (0.12, 4.12)	.8
Monitor position	1.86 (0.39, 9.47)	.4	1.66 (0.40, 7.18)	.5	2.06 (0.64, 6.91)	.2
Patient habitus	1.00 (0.31, 3.26)	.9	0.38 (0.11, 1.18)	.11	3.19 (0.79, 13.7)	.1
Glove number	0.84 (0.26, 2.68)	.77	0.41 (0.10, 1.37)	.17	1.48 (0.36, 40)	.5
Switching holding endoscope	0.76 (0.03, 20.57)	.85	0.58 (0.02, 15.62)	.71	0.36 (0.08, 1.32)	.152
**Nostril and endoscopic tool position**	1.00 (0.13, 9.39)	1	**0.27 (0.07, 0.89)**	**.037**	0.00 (0.00, 6.03)	.99
**Operate on same side as co‐surgeon**	0.68 (0.21, 2.20)	.52	**0.25 (0.07, 0.82)**	**.026**	0.00 (0.00, 6.03)	.99

Abbreviations: CI, confidence interval; OR, odds ratio.

### Musculoskeletal Pain Experienced by EESBs—Academic and Private Physicians Stratified Analysis

Academic and private EESBs indicated that they experience musculoskeletal pain in similar areas (*P *> .05) except in the lower back/L‐spine, which was more commonly reported by academic physicians and may be due to the small sample size (*P* = .002). Similar to the whole cohort analysis, dominant hand, operative glove numbers, switching endoscope holding during cases, foot pedal position, fixed posture with endoscope holding, monitor position, and patient habitus did not impact musculoskeletal strain for academic or private physicians (*P* > .05). Prolonged standing (OR: 6.2; CI: 1.38, 39.6; *P* = .029) and manipulation of endoscopic instruments (OR: 9.9; CI: 1.60, 102; *P* = .025) were associated with significantly increased risk of experiencing neck/C‐spine pain in academic physicians. This relationship was not seen in the private physician cohort. Academic EESBs that operate on the same side during ESBS were less likely to experience musculoskeletal strain (OR: 0.18; CI: 0.03, 0.89; *P* = .043) in general and L‐spine pain (OR: 0.19; CI: 0.04, 0.83; *P* = .036) compared to those who operate on the opposite side of the table. Moreover, academic EESBs that operate with the rhinologist at the head of the patient and the neurosurgeon on the side of the patient had similar overall musculoskeletal strain and L‐spine strain as those that operate on the opposite side of the table (*P *> .05) (Operative Information Question Number 7). This relationship was not seen with private EESBs. During endoscopic resection, academic EESBs who place 1 instrument in the same nostril below the endoscope and other instruments in the opposite nostril from the endoscope were less likely to experience musculoskeletal strain than academic EESBs that place all of their instruments in the opposite nostril (OR: 0.09; CI: 0.00, 0.58; *P *= .032). This relationship was not seen in private EESBs. Factors that influence musculoskeletal strain in academic physicians are seen in Table 5. There were no significant factors that influenced musculoskeletal strain in private practice EESBs.

**Table 5:** Analysis of Factors That Influence Musculoskeletal Strain for Academic EESBs

Strain						
	Neck/C‐Spine		Low back/L‐Spine		MSK strain > 4/10	
Variable	Odds ratio (CI)	*P*‐value	Odds ratio (CI)	*P*‐value	Odds ratio (CI)	*P*‐value
Prolonged standing	**6.20 (1.38,39.60)**	**.029**	1.13 (0.29, 4.28)	.9	1.23 (0.32, 4.88)	.8
Manipulation of endoscopic instruments	**9.97 (1.60, 102)**	**.025**	0.21 (0.03, 1.04)	.067	1.08 (0.18, 5.51)	.9
Foot pedal position	1.06 (0.13, 8.26)	.9	1.29 (0.20, 8.54)	.8	0.57 (0.06, 3.64)	.6
Monitor position	0.77 (0.08, 6.10)	.8	2.14 (0.59, 8.41)	.3	2.27 (0.60, 9.31)	.2
Patient habitus	2.49 (0.50, 15.0)	.3	3.13 (0.53, 24.5)	.2	1.90 (0.31, 10.9)	.5
Glove number	0.98 (0.24, 3.82)	.97	0.29 (0.06, 1.21)	.1	1.47 (0.33, 6.39)	.6
Switching holding endoscope	5 (0, 6)	.99	0.23 (0.04, 1.00)	.06	0.39 (0.05, 1.82)	.28
Nostril and endoscopic tool position	1.0 (0.09, 11.32)	1	**0.09 (0.00, 0.58)**	**.032**	0.65 (0.15, 3.02)	.57
Operate on same side as co‐surgeon	0.62 (0.15, 2.52)	.5	**0.19 (0.04, 0.83)**	**.036**	**0.18 (0.03, 0.89)**	**.04**

Abbreviations: CI, confidence interval; OR, odds ratio.

### Musculoskeletal Pain Experienced by EESBs Comparing Neurosurgeons and Rhinologists

Both rhinologists and neurosurgeons experience musculoskeletal strain in similar areas (*P* > .05). Similar to the entire cohort analysis, dominant hand, operative glove amount, switching endoscope holding during cases, fixed posture with endoscope holding, and monitor position did not impact musculoskeletal strain for neurosurgeons or rhinologists (*P* > .05). Neurosurgeons that use their right hand to manipulate the endoscope during endoscopic cases have significantly less musculoskeletal strain than those that use either hand (OR: 0.05; CI: 0.001‐0.525; *P* = .027). Additionally, neurosurgeons that operate on the same side as rhinologists during endoscopic skull base surgeries have less musculoskeletal and C‐spine strain than those that operate on opposite sides of the table (OR: 0.14; CI: 0.02‐0.85; *P* = .04). All factors that influence musculoskeletal strain in neurosurgeons are seen in Table 6. There were no significant factors that influenced musculoskeletal strain in rhinologists.

**Table 6:** Analysis of Factors That Influence Musculoskeletal Strain for Neurosurgeons

Strain						
	Neck/C‐Spine		Low back/L‐Spine		MSK strain > 4/10	
Variable	Odds ratio (CI)	*P*‐value	Odds ratio (CI)	*P*‐value	Odds ratio (CI)	*P*‐value
Prolonged standing	0 (0, 40)	.99	1.26 (0.08, 23.00)	.9	0.75 (0.02, 24.66)	.86
Manipulation of endoscopic instruments	0.92 (0.02, 49.1)	.99	0.07 (0.00, 1.05)	.1	0.07 (0.00, 1.05)	.1
Foot pedal position	0 (0, 40)	.99	1.07 (0.03, 30.30)	.99	21 (0.67, 3128)	.15
Monitor position	1.17 (0.03, 49.0)	.99	6.67 (0.17, 334)	.3	3.66 (0.09, 390)	.5
**Patient habitus**	0.79 (0.09, 6.70)	.8	**0.06 (0.01, 0.42)**	**.01**	0.55 (0.086, 3.679)	.553
Glove number	0.66 (0.15, 2.83)	.57	0.35 (0.07, 1.57)	.18	0.36 (0.06, 1.82)	.234
Switching holding endoscope	0 (0,40)	.99	1.50 (0.17, 15.55)	.71	0 (0, 40)	.99
Nostril and endoscopic tool position	0.60 (0.05, 7.33)	.68	0.27 (0.02, 2.82)	.29	0.55 (0.086, 3.679)	.553
**Operate on same side as co‐surgeon**	**0.14 (0.02, 0.85)**	**.04**	1.50 (0.17, 15.55)	.72	**0.14 (0.016, 0.888)**	**.048**

### Ergonomic Awareness

77% of respondents stated that their musculoskeletal strain does not affect their work as a surgeon and 68% of respondents stated that the strain does not affect their life outside of work. While 60% of respondents agreed or strongly agreed that they are concerned about their overall musculoskeletal health affecting their practice, 75% of respondents indicated their institution does not provide ergonomic measures. 89% of respondents agreed or strongly agreed that they would be open to trying interventions and 78% of respondents agreed or strongly agreed that ergonomic interventions have the potential to improve musculoskeletal strain in the operating room. The most common ergonomic practices implemented by surgeons currently include microbreaks (48%) and stretching (45%) with microbreaks providing significant improvement in strain (OR: 5.6; CI: 1.1‐36.0; *P* = .04).

## Discussion

The incorporation of endoscopic surgery into practice for rhinologists and neurosurgeons has been revolutionary in skull base surgery for patients. While endoscopic surgery has transformed the lives of many patients, the prevalence of work‐related musculoskeletal strain is higher in endoscopists compared to other surgical and medical specialties.^9^, ^11^, ^12^ This survey‐based study aimed to obtain a current assessment of self‐reported musculoskeletal stain among endoscopic skull base surgeons to better characterize which factors influence pain and develop prevention techniques. The results of the survey demonstrated that endoscopic sinus and skull base surgeons experience work‐related musculoskeletal strain in the neck (50%) followed by the L‐Spine (42%). A 2024 meta‐analysis investigating contributing factors to ergonomic strain for skull base surgeons demonstrated a similar distribution of musculoskeletal strain with the neck, lumbar spine, and thoracic spine as the most involved sites.^11^

This study compared multiple endoscopic positioning techniques to assess the impact of operating room set up on musculoskeletal strain. We compared 3 primary set ups: (A) 2 surgeons working on the same side of the patient with 1 monitor, (B) 1 surgeon at the head of the patient and 1 at the side of the patient with 2 monitors, (C) 2 surgeons on opposite sides of the patient with 2 monitors. Our results showed that surgeons who operate on the same side of the patient are less likely to have musculoskeletal strain than surgeons who operate on opposite sides of the patient. We also found that surgeons who operate with 1 surgeon at the head of the patient and 1 surgeon on the side of the patient have similar strain to surgeons that operate on opposite sides of the patient. This trend was seen specifically in academic physicians and neurosurgeons who responded to the survey. Mattogno et al, demonstrated that 64% of their respondents operated on the same side of the table and identified potential ergonomic issues with this method.^10^ Mattogno et al, states that if the primary surgeon is not holding the endoscope during tumor removal, the cervicodorsal spine is rotated and/or flexed to achieve a symmetric use of arms.^10^ Mattogno et al, also states that while a minority of their respondents place surgeons on opposite sites, there may be an adjunctive physical stress on both upper arms, cervical, and lumbar spines for both surgeons.^10^ Our study builds on these observations by attempting to provide quantitative and objective data to surgeon positioning in the operating room with musculoskeletal strain. Two previous observational studies by Maniam et al, have also assessed the impact of surgeon positioning on musculoskeletal strain and have shown that the most ergonomically favorable positioning is for surgeons to stand on opposite sides with 2 monitor screens.^13^, ^14^ Maniem et al, also demonstrated the least favorable position involved only 1 monitor screen or both surgeons standing on the same side.^13^, ^14^ While both studies differ in surgeon positioning that increases ergonomic strain, the optimal arrangement of surgeons in the operating room will ultimately depend on the comfort of each individual surgeon, the size of the operating room, and the extent of the surgical set up needed for each case.^15^ Additionally, along with optimal set up, factors including wearing lead protection and the number of sinus and skull base cases may impact ergonomic strain in the operating room. Future work with objective data is needed as multiple studies have now shown that the positioning of surgeons in the operating room can influence musculoskeletal strain.

This study further investigated the impact of instrument and endoscope positioning while operating. Historically, a uninarial approach was favored and considered to be less traumatic than a binarial approach. However, the uninarial approach limits the number of instruments that can be used while operating.^15^ This study found that surgeons who place 1 instrument in the same nostril below the endoscope and instruments in the opposite nostril were less likely to experience ergonomic strain compared to surgeons who place all instruments in the opposite nostril from the endoscope. While this study did not specifically ask which instruments should be placed where, future research regarding specific tools with objective data can help better understand these preliminary findings. Moreover, this study found that operating room and instrument set up factors including monitor position and foot pedal position did not impact musculoskeletal strain Previous studies have demonstrated that monitor position can impact musculoskeletal strain and that the optimal position for the monitor is frontal and at eye level.^16^, ^17^ The results shown in this study likely reflect an adoption of optimal monitor heights and positioning across institutions decreasing musculoskeletal strain. Additionally, this study did not identify an association between average hours operating a week and musculoskeletal strain experienced by EESBs. The results, however, observe that prolonged standing increases cervical spine pain in EESBs. Previous studies have suggested that work related musculoskeletal strain positively correlates with the number of procedures performed and that cumulative years performing endoscopic tasks predicts worsening neck and arm discomfort.^5^ This was not seen in this study population and future work is needed to identify the optimal number of cases performed daily to minimize strain. This study also explored factors including dominant hand, switching out holding the endoscope during cases, foot pedal position, manipulation of endoscopic instruments glove size, and maintaining a fixed posture while holding the endoscope and found no significant impact on musculoskeletal strain for any body part. Prior studies have not examined these effects in endoscopic surgeons and future studies with objective measurements would help clarify additional factors that may contribute to strain in this physician population.

The results of this study indicate that academic and private practice physicians experience musculoskeletal strain similarly, however there were significantly fewer respondent private practice physicians than academic physicians Thus, though certain factors that influence musculoskeletal strain in academic physicians were not seen in private practice physicians, the results should be interpreted cautiously given the apparent limitation in sample size that may reduce the statistical power in any sub analysis involving only private practice physicians. Future work with larger sample sizes of private practice EESBs is needed to determine which factors influence musculoskeletal strain. Additionally, the results of this study suggested that musculoskeletal strain experienced by academic physicians is influenced by prolonged standing. This was not seen in the private practice physician cohort in this study. Academic physicians routinely operate with residents and fellows and may not be standing for the entire case load that private physicians would be. Further studies are needed to better understand the impact of prolonged standing on musculoskeletal strain while accounting for operating with residents and fellows for academic physicians versus private physicians.

Although most EESBs in this study did not find that musculoskeletal strain impacts their work as a surgeon, most are concerned about its potential impact. Respondents also indicated they would be open to trying interventions to help decrease strain, but most centers do not provide ergonomic measures. Similar to previously published studies, microbreaks significantly improve musculoskeletal strain in the operating room. Incorporating microbreaks early into surgical training can help reduce work‐related strain for EESBs.^18^, ^19^, ^20^ Further studies objectively assessing factors that specifically impact musculoskeletal strain are essential for EESBs.

### Limitations

The limitations of this study are acknowledged. Although the response rate is comparable to other surveys distributed to NASBS members, only a small proportion of NASBS members responded to the survey, potentially resulting in a sampling bias.^9^, ^21^, ^22^ Additionally, since the questionnaire was lengthy, surgeons that are bothered by their musculoskeletal strain are more likely to respond. While most respondents were largely reflective of academic centers and an equal number of neurosurgeons and rhinologists responded, geographic differences and setting volume, which can impact strain, were not assessed. The results of this study are largely reflective academic physicians, who comprise the vast majority of respondents to this survey. Additional studies including more private practice EESBs would help better understand which factors influence strain amongst them and identify any apparent differences between academic and private practices. Future studies should reflect if different positions (same side, opposite side, or rhinologists at the head) differ for musculoskeletal strain for neurosurgeons versus rhinologists within the same team as well as the impact dictation versus typing operative reports has on musculoskeletal strain.

## Conclusions

Work‐related musculoskeletal strain is highly prevalent amongst EESBs. Self‐reported factors including surgeon position in the operating room, prolonged standing, and endoscopic instrument position can increase musculoskeletal strain. Future work with objective data is needed to better understand the impact of ergonomic strain on EESBs.

## Author Contributions

**Shreya Mandloi**, MD, study design, data collection, statistical analysis, manuscript writing and editing; **Alexander Duffy**, MD, study design, data collection, manuscript writing and editing; **Praneet Kaki**, BS, study design, data collection, statistical analysis, manuscript writing and editing; **Elina M. Toskala**, MD, PhD, MBA, manuscript writing and editing; **Marc Rosen**, MD, manuscript writing and editing; **Christopher Farrell**, MD, manuscript writing and editing; **James Evans**, MD, manuscript writing and editing; **Gurston G. Nyquist**, MD, manuscript writing and editing; **Mindy Rabinowitz**, MD, study design, manuscript writing and editing.

## Disclosures

Accepted as an Oral Presentation at North American Skull Base 2025, New Orleans, Louisiana.

### Competing interests

None.

### Funding source

None.

## Supporting information

supporting information.
