# Supplementary material for: Ergonomic Trends in Endoscopic Skull‐Base Surgeons: A Survey to the North American Skull Base Society
Source: OTO Open. 2026 Mar 9;10(1):e70220. doi: 10.1002/oto2.70220 (PMC12969492; doi:10.1002/oto2.70220)
Supplement: Supplementary file 1 — supporting information. [file OTO2-10-e70220-s001.docx]

***QUESTIONNAIRE:***

***Demographics, General Information***

1. What is your age (years)?
   1. Open-ended
2. What gender do you identify with?
   1. Male
   2. Female
   3. Other/nonbinary
   4. Choose not to answer
3. Please enter your approximate **height (inches)** and **weight (lbs)**
   1. Open-ended
4. How many years of experience do you have in practice (since completing residency/fellowship)?
   1. Open-ended
5. What are your average operating hours per week?
   1. Likert Sliding Scale – 0 to 40+
6. What percentage of your operative time is dedicated strictly to endoscopic skull base cases?
   1. Sliding Scale – Percentages, 0-100%
7. What is your specialty?
   1. Neurosurgeon
   2. Rhinologist
8. Where is your primary place of practice?
   1. Academics
   2. Private
   3. Other: ___________
9. Do you routinely operate with residents or fellows?
   1. Yes
   2. No
10. Do you routinely exercise or perform strenuous activities outside of work? Check any that apply.
    1. Cardio
    2. Weightlifting
    3. Group Fitness
    4. Other: _________
    5. None
11. How would you describe your current musculoskeletal health?
    1. Very Poor
    2. Poor
    3. Average
    4. Good
    5. Excellent

***Operative Information***

1. What is your dominant hand?
   1. Right-handed
   2. Left-handed
   3. Ambidextrous
2. Do you operate with one glove or two gloves on each hand?
   1. One glove
   2. Two gloves
3. Top Glove Size
   1. Open-ended
4. What percentage of cases are you using an endoscope?
   1. < 10%
   2. 10-24%
   3. 25-50%
   4. 51-75%
   5. >75%
5. Which hand do you use to manipulate endoscopic instruments during the endoscopic approach for skull-base surgery?
   1. Right hand
   2. Left hand
   3. Either hand
   4. I do not perform the approach portion of surgery
6. What hand do you use to control/manipulate the **endoscope** during **dual surgeon** endoscopic skull base surgery (i.e. when both surgeons are operating simultaneously)?
   1. Right
   2. Left
   3. Both
   4. I do not manipulate the endoscope during this phase
7. Which of the following pictures most accurately depicts your OR setup during the dual surgeon portion of endoscopic skull base surgery?
   1.
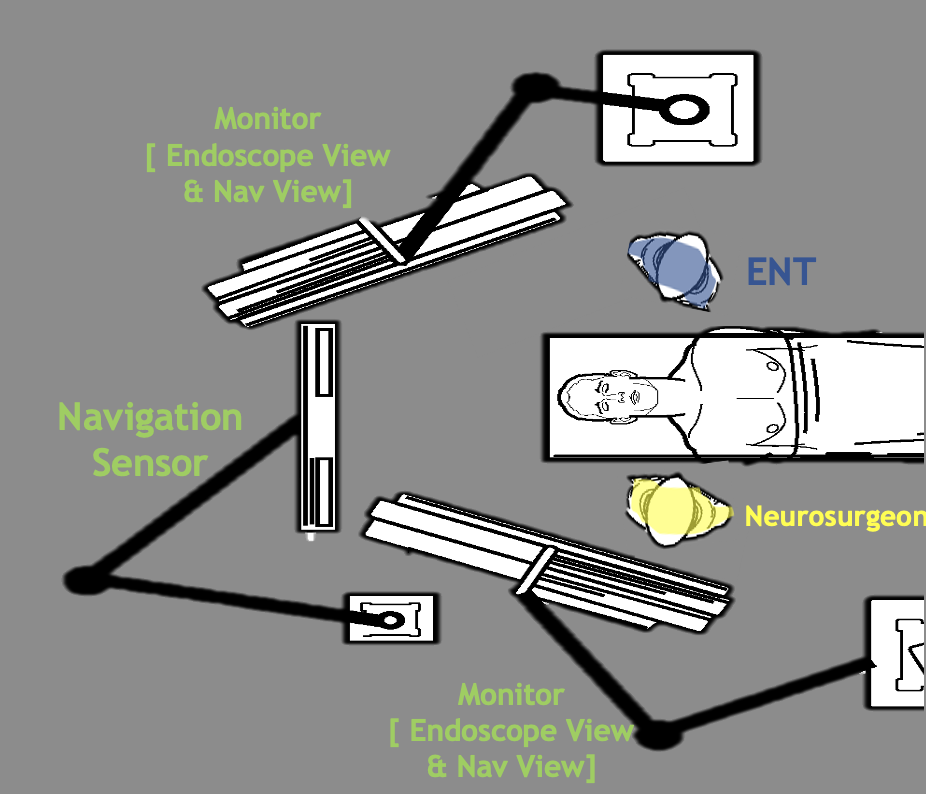

   2.
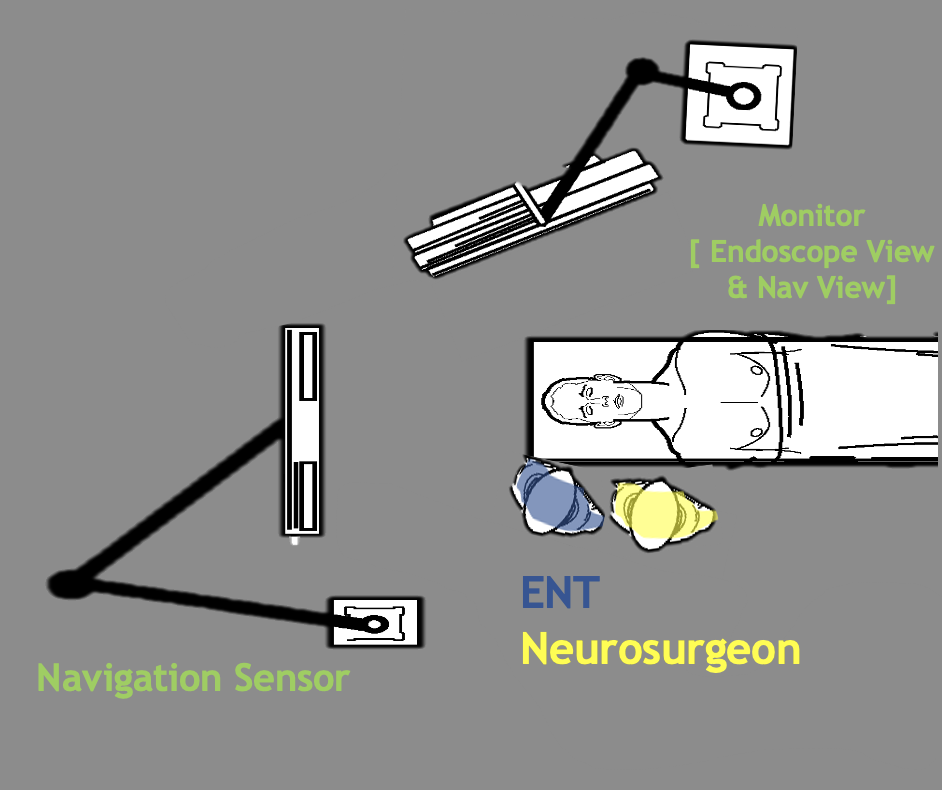

   3.
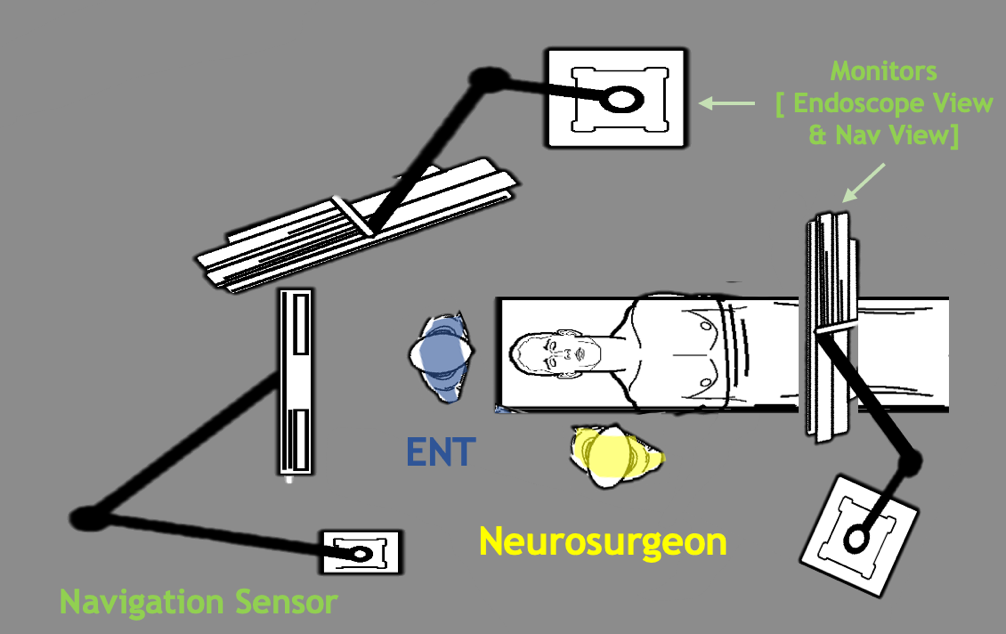

   4. Other / Not depicted
8. During neurosurgical resection, the endoscopic instruments typically are used:
   1. In the same nostril, atop the endoscope
   2. In the same nostril, below the endoscope
   3. In the opposite nostril from the endoscope
   4. One instrument in the same nostril atop the endoscope and one instrument in the opposite nostril from the endoscope
   5. One instrument in the same nostril below the endoscope and one instrument in the opposite nostril from the endoscope
9. Please select the most accurate response - During the **dual surgeon** portion of endoscopic cases, the **neurosurgeon** utilizes
   1. Both hands manipulate operative instruments while another person holds the endoscope
   2. Both hands manipulate operative instruments while a fixed scope holder stabilizes the endoscope.
   3. One hand to manipulate operative instruments and one hand to hold the endoscope
10. In a typical **endoscopic sinus surgery case**, what percentage of the time are you physically operating (i.e. not a fellow or resident)?
    1. < 10%
    2. 10-24%
    3. 25-50%
    4. 51-75%
    5. >75%
11. In a typical **endoscopic skull base** case, what percentage of the time are you physically operating (i.e. not a fellow or resident)?
    1. < 10%
    2. 10-24%
    3. 25-50%
    4. 51-75%
    5. >75%
12. Please describe the set-up for holding scope during the neurosurgical portion of endoscopic skull-base surgeries
    1. A resident or fellow holds the scope
    2. An attending (myself) holds the scope
    3. An attending (other than myself) holds the scope
    4. A fixed scope holder is used
13. Please describe the set up for holding the scope during the neurosurgical portion of endoscopic skull-base surgeries
    1. A resident or fellow switches off with the attending to hold the scope at a fixed point in time
    2. A resident or fellow switches off with another resident or fellow at a fixed point in time
    3. An attending switches off with another attending at a fixed point in time
    4. A resident or fellow holds the scope throughout the case without switching off
    5. An attending holds the scope throughout the case without switching off
    6. I do not hold the scope. I use a fixed scope holder.

***Symptomatology***

1. Which body parts are significantly affected (> 4 out of 10) by your participation in surgery? (Check all that apply)
   1. Neck / C-spine
   2. Shoulders
   3. Upper Back
   4. Lower Back / L-spine
   5. Dominant Upper Extremity
   6. Dominant Wrist
   7. Dominant Hands-Fingers
   8. Lower Extremities
   9. Feet
   10. Other: __________
2. If you selected any body parts in the prior question, how would you describe your symptoms? (Select all that apply)
   1. Pain
   2. Fatigue
   3. Stiffness
   4. Paresthesia/Numbness
   5. Weakness
   6. Option to skip question
3. Please rate the average severity of your musculoskeletal symptoms
   1. Likert Scale 0-10
4. How often do you experience musculoskeletal discomfort related to surgery?
   1. < 25% of the time
   2. 25-50% of the time
   3. 51-75% of the time
   4. >75% of the time
5. Which aspects of endoscopic sinus/skull-base surgery aggravate your musculoskeletal symptoms? (Check all that apply)
   1. Prolonged standing
   2. Manipulation of endoscopic instruments
   3. Foot pedal position
   4. Fixed posture during scope-holding
   5. Monitor position
   6. Patient habitus
   7. Other: ___________
6. Which activities/conditions outside of work do you attribute to your musculoskeletal symptoms, if any? (Check all that apply)
   1. Workout regimen
   2. Strenuous hobby
   3. Inactivity
   4. Medical condition
   5. Other: _______
7. How severely has your discomfort/pain affected your ability to work as a surgeon?
   1. Not at all
   2. Somewhat
   3. Significantly
8. How severely has your discomfort/pain affected your life outside of work?
   1. Not at all
   2. Somewhat
   3. Significantly
9. Describe how your symptoms have impacted your practice. (Check all that apply)
   1. Taken days off
   2. Increased operative time due to discomfort
   3. Reduced operative case numbers
   4. Reduced office hours/clinic following an operative day
   5. None of the above
   6. Other: _______
10. Which type(s) of treatment have you pursued for your discomfort or pain attributable to work in the OR?
    1. Systemic analgesics (Over-the-counter)
    2. Systemic analgesics (prescription)
    3. Topical treatments
    4. Massage, chiropractor, or acupuncture therapy
    5. None
    6. Other: ______
11. How often do you utilize these treatments?
    1. Never / I do not use any treatments
    2. Once weekly
    3. More than once weekly
    4. Daily
12. Does your department or institution provide ergonomic measures or recommendations?
    1. Yes
    2. No
    3. Not sure
13. What current ergonomic practices do you employ in your operative practice?
    1. Ergonomic furniture (floor mats, medicine ball, etc.)
    2. Ergonomic apparel (posture devices, shoe inserts, compression socks, etc.)
    3. Micro breaks
    4. Stretching between cases
    5. None
14. In the operative room, do you have any of the following ergonomic pieces of furniture?
    1. Floor mat
    2. Medicine Ball
    3. Seat cushion
    4. None

***Please rate your agreement with the following:***

1. I have learned to accept and live with the discomfort brought on from operating
   1. Strongly Disagree
   2. Disagree
   3. Neutral
   4. Agree
   5. Strongly Agree
2. I am concerned about my overall musculoskeletal health suffering from my practice
   1. Strongly Disagree
   2. Disagree
   3. Neutral
   4. Agree
   5. Strongly Agree
3. I believe that ergonomic practices have the potential to improve my overall musculoskeletal comfort in the operating room
   1. Strongly Disagree
   2. Disagree
   3. Neutral
   4. Agree
   5. Strongly Agree
4. I would be open to trying interventions to improve my musculoskeletal health in the operating room
   1. Strongly Disagree
   2. Disagree
   3. Neutral
   4. Agree
   5. Strongly Agree
5. Ergonomic strain has negatively affected my surgical career
   1. Strongly Disagree
   2. Disagree
   3. Neutral
   4. Agree
   5. Strongly Agree
